# Chemical Composition, Production of Secondary Metabolites and Antioxidant Activity in Coffee Cultivars Susceptible and Partially Resistant to Bacterial Halo Blight

**DOI:** 10.3390/plants10091915

**Published:** 2021-09-15

**Authors:** Joyce Alves Goulart da Silva, Mário Lúcio Vilela de Resende, Ingridy Simone Ribeiro, Adriene Ribeiro Lima, Luiz Roberto Marques Albuquerque, Ana Cristina Andrade Monteiro, Matheus Henrique Brito Pereira, Deila Magna dos Santos Botelho

**Affiliations:** 1 Universidade Federal de Lavras, UFLA, Departamento de Fitopatologia, Lavras, MG 37200-900, Brazil; joycegoulart@hotmail.com (J.A.G.d.S.); mlucio@ufla.br (M.L.V.d.R.); monteiroaca@yahoo.com.br (A.C.A.M.); matheus.pereira6@estudante.ufla.br (M.H.B.P.); 2 Instituto Federal do Sul de Minas Gerais (IFSULDEMINAS), Campus Muzambinho, Muzambinho, MG 37890-000, Brazil; ingridy.ribeiro@muz.ifsuldeminas.edu.br; 3 Universidade Federal Fluminense (UFF), Faculdade de Farmácia, Departamento de Bromatologia, Niterói, RJ 24241-000, Brazil; adrienelima@id.uff.br; 4 Universidade Federal dos Vales do Jequitinhonha e Mucuri (UFVJM), Janaúba, MG 39440-000, Brazil; luiz.albuquerque@ufvjm.edu.br

**Keywords:** *Coffea arabica*, *Pseudomonas syringae* pv. *garcae*, chlorogenic acid, phenolic compounds

## Abstract

Coffee production is one of the main agricultural activities in Brazil, and several coffee cultivars with disease resistance have already been developed. The secondary metabolites produced by plants are closely associated with defense strategies, and the resistance of coffee cultivars to bacterial halo blight (BHB) can be related to these compounds. Therefore, this study aims to compare a partially resistant coffee cultivar (Iapar-59) and a susceptible cultivar (Mundo Novo 376/4) to BHB (*Pseudomonas syringae* pv*. garcae*) in relation to the chemical composition and antioxidant activity of the leaf extracts. In addition, this study determined the total phenolic and flavonoid contents and phenolic profiles of the Iapar-59 leaf extracts of plants inoculated with *P. syringae* pv. *garcae*. The Iapar-59 extract showed a higher content of phenolic compounds and flavonoids than the Mundo Novo 376/4 extract. Both cultivars contained gallic, chlorogenic and caffeic acids; however, the highest contents were quantified in the Iapar-59 cultivar. The leaf extracts from the Iapar-59 cultivar exhibited higher antioxidant activity. Higher concentrations of gallic, caffeic and chlorogenic acids and the presence of vanillin were detected in the extract of cultivar Iapar-59 inoculated with *P. syringae* pv. *garcae*.

## 1. Introduction

Plants produce large amounts of organic compounds without a direct function in their growth and development. These substances are secondary metabolites and are strongly involved in the interaction between plants and pathogens [1]. Phenolic compounds including phenylpropanoids and flavonoids are important secondary metabolites synthesized by plants. Phenolic compounds are toxic to pathogens and are rapidly produced and accumulate after infection, particularly in resistant varieties. Chlorogenic, caffeic and gallic acids are examples of some of these compounds. [1,2,3]. Flavonoids are involved in plant protection against pathogens, and defense-related flavonoids can be divided into preformed (basal resistance) or induced by stress, biotic/abiotic [4]. The phytobacterium *Pseudomonas syringae* trigger the synthesis of flavonoids in plants [5].

Studies demonstrating the induction of defense genes by sugars in the absence of pathogens elucidated the mechanism linking the carbohydrate metabolism with defense responses. Sugars provide energy and structural material for defense responses in plants. Moreover, they may also act as signal molecules that interact with the hormonal signaling network regulating the plant immune system [6,7,8].

Plants have many bioactive compounds with high antioxidant activity, which indicates a plant’s ability to scavenge free radicals [9,10], and this activity is related to the severity of diseases [11]. The antioxidant activity, flavonoids, phenolic compounds and sugars in plants can vary according to species, cultivar, resistance level to diseases, and are also dependent on the plant pathogen/pest interaction as *Oidium xanthami* in *Xanthium strumarium* [11], *Leucoptera coffeella* and *Hemileia vastatrix* in coffee [12,13], and *Pseudomonas syringae* in *Arabidopsis thaliana*, kiwi, and tomato [5,14,15,16].

Coffee is a crop of significant economic importance in many countries including Brazil, and several factors limit coffee production, including fungal and bacterial diseases [17]. The bacterial halo blight of coffee (BHB) caused by *Pseudomonas syringae* pv*. garcae* [18,19], is a disease that has caused losses in nurseries and fields established in regions subjected to strong winds, mild temperatures, and frequent and well-distributed rains [17,20]. Among the coffee (*Coffea arabica* L.) cultivars planted in Brazil, the Mundo Novo 376/4 cultivar corresponds to almost half of the planted area. This cultivar is susceptible to main coffee diseases, including BHB [21,22]. The Iapar-59 cultivar has an intermediate level of resistance to BHB [23,24] and is resistant to coffee leaf rust (*Hemileia vastatrix*) [23,25]. Coffee leaves have constitutive defense substances such as phenolic compounds [12,13], and information on the chemical composition of coffee cultivars under basal defense induction conditions against *Pseudomonas syringae* pv. *garcae* can help understand the mechanisms that act during pathogenesis and may assist resistance breeding programs to coffee diseases.

Therefore, this study aims to determine the difference in the content of secondary metabolites and antioxidant activities of coffee leaf extracts from Mundo Novo 376/4 and Iapar-59 cultivars. Additionally, this study determined the total phenolic and flavonoid contents and phenolic profiles of Iapar-59 leaf extracts of plants inoculated and non-inoculated with *P. syringae* pv. *garcae*.

## 2. Results

### 2.1. Chemical Composition and Antioxidant Activity of Coffee Leaf Extracts from Mundo Novo 376/4 and Iapar-59 Cultivars

The cultivar Iapar-59 showed the highest phenolic compounds and flavonoid contents, differing significantly from the Mundo Novo 376/4 cultivar (Table 1). The phenolic compounds and flavonoids contents were 66% and 53% higher, respectively, in leaf extracts from Iapar-59.

The phenolic profile quantified by HPLC identified gallic, caffeic and chlorogenic acids in the extracts of both the Iapar-59 and Mundo Novo cultivars; however, the Iapar-59 cultivar showed a higher concentration of gallic and caffeic acid than the Mundo Novo cultivar (Table 1).

**Figure 1:**
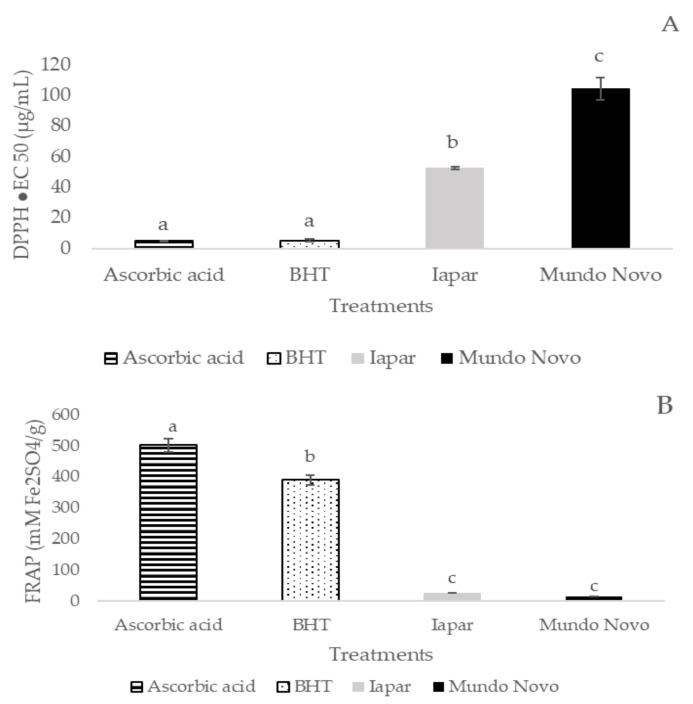
Antioxidant activities of the Iapar-59 and Mundo Novo 376/4 cultivars and the standards (ascorbic acid and butylated hydroxytoluene: BHT) by the methods of free radical scavenging activity: DPPH (µg/mL) (**A**), and ferric reducing antioxidant power: FRAP (mM Fe_2_SO_4_/g) (**B**). Bars followed by the same lowercase letter do not differ statistically by Tukey test (*p* < 0.05) (n = 6 biological replicates).

Coffee leaf extracts from Mundo Novo and Iapar-59 showed lower antioxidant activity than the standards used (Figure 1A,B). However, when the studied cultivars were compared, Iapar-59 showed higher antioxidant activity (quantified by the DPPH method) than the Mundo Novo 376/4 cultivar. The antioxidant activity (quantified by the FRAP method), was similar in both cultivars (Figure 1A,B).

### 2.2. Production of Secondary Metabolites in Iapar-59 Coffee Leaves Inoculated with P. syringae pv. garcae

Extracts from intact seedlings (non-inoculated with *P. syringae* pv. *garcae*) showed the highest content of total phenolic compounds, reducing sugars and flavonoids (Table 2). Higher concentrations of gallic, caffeic and chlorogenic acids and the presence of vanillin were detected in the extract of Iapar-59 inoculated with *P. syringae* pv. *garcae* than in the control samples (non-inoculated plants) (Table 2).

The severity of BHB in Mundo Novo and Iapar-59 cultivars was 16.0% and 6% of the diseased leaf area, respectively.

## 3. Discussion

The Mundo Novo and Iapar-59 cultivars showed variation in disease severity, as reported by Andreazi et al. [22], who evaluated the resistance to BHB in trials performed under field conditions. According to the authors, the disease severity in Mundo Novo was five to ten times more than in Iapar-59.

Coffee leaf extracts from the Iapar-59 cultivar showed the highest phenolic compounds, flavonoids, caffeic acid and gallic acid. Flavonoids may be constitutively synthesized; however, their biosynthesis is often enhanced under the influence of stress including pathogens [4]. According to Treutter [4] phenolic compounds, including flavonoids, often accumulate in specialized cells and can be infused into attacked tissues. Such leaching is possibly involved in common mechanisms of pathogen defense such as programmed cell death and hypersensitive response. This action mode associated with the higher phenolic compound and flavonoids can explain the moderate resistance of Iapar-59 to BHB and its high level of resistance to coffee rust [23,24,25]. Caffeic acid is involved in the synthesis of lignin, and gallic acid has antioxidant and antimicrobial properties [3,26]. Phenolic compounds and lignin are produced and may accumulate as chemical and physical defenses against pathogens [2,3].

The aqueous extracts from both tested cultivars showed lower activity than the standard ascorbic acid and butylated hydroxytoluene (BHT). The highest antioxidant activity observed in the Iapar-59 cultivar correlated with the higher content of total phenolics. According to Ngamsuk et al. [27], the DPPH radical scavenging activity of coffee leaves is significantly correlated with total phenolic content. The cultivar Iapar-59 (cross between Villa Sarchi and Timor hybrid CIFC 832/2), has in its genealogy genes from *Coffea canephora* due to the Timor hybrid (natural interspecific cross between *C. arabica* and *C. canephora*) [28,29]. *C. canephora* has a higher soluble solid content, caffeine and chlorogenic acid [30]. Therefore, the results found higher phenolic compounds content and antioxidant activity in Iapar-59, which may be correlated with the genotypic origin of this cultivar.

The inoculation with *P. syringae* pv. *garcae* altered the contents of compounds evaluated. Studies have reported the increased accumulation of phenolic compounds in olive trees due to infection by *Pseudomonas savastanoi* pv*. savastanoi* [31,32]. Tomato plants respond to *Pseudomonas syringae* pv *tomato* DC3000 infection by producing flavonoids and other phenolic compounds [5]. However, in the present study, the total phenolic compounds, flavonoids and reducing sugars content decreased upon infection by *P. syringae* pv. *garcae*. The decrease in sugars was observed by Li et al. [15] in kiwi leaves infected with *Pseudomonas syringae* pv. *actinidae* (Psa). According to authors the reduced content of sugars indicated that Psa infection directly or indirectly inhibited carbon fixation and sugar metabolism.

Plant pathogens can actively suppress the expression of plant defense reactions during successful infection [33]. Resistant and susceptible olive cultivars inoculated with *Verticillium dahliae* showed a decrease in total phenolics. According to the authors, this decrease was associated with the fungal DNA level in the susceptible cultivar [34], indicating the influence of the infection process on the reduction in the synthesis of these compounds in the plant. Then, our results suggested that P. *syringae* pv. *garcae* may induce reduction in the compounds (sugars, phenolic compounds and flavonoids) in coffee seedlings during the infection process as observed in other pathosystems [15,34]. Higher concentrations of gallic, caffeic, and chlorogenic acids and the presence of vanillin were detected in the Iapar-59 cultivar inoculated with *P. syringae* pv. *garcae* than in the non-inoculated ones. Vanillin was detected only in the partially resistant Iapar-59 inoculated plants and may be considered a putative phytoalexin in coffee leaves, since it was not detected in non-inoculated plants. At present, no phytoalexin-like compound has been confirmed in coffee; however, the accumulation of caffeic, chlorogenic, and gallic acids has been reported as a defense response to phytopathogens such as *Hemileia vastatrix* in coffee [35], *Xylella fastidiosa* in grape plants [36], *Pseudomonas syringae* pv. *tomato* DC3000 in tomato [37], and *Fusarium* in cucumber [38].

In summary, this study provides information regarding the chemical composition of coffee cultivars under basal defense induction conditions against *Pseudomonas syringae* pv. *garcae* as well as the detection for the first time of vanillin in leaves of a partially resistant cultivar to BHB. These results can help to understand the mechanisms that act during pathogenesis and may assist resistance breeding programs regarding coffee diseases.

## 4. Material and Methods

### 4.1. Chemical Composition and Antioxidant Activity of Coffee Leaf Extracts from Mundo Novo 376/4 and Iapar-59 Cultivars

#### 4.1.1. Preparation of Coffee Seedlings

Coffee seedlings from the cultivars Iapar-59 and Mundo Novo 376/4 with four pairs of fully expanded leaves were grown in black polyethylene bags (0.11 × 0.20 m) containing a substrate composed of soil: sand: substrate for vegetables (Plantmax) at a 2:1:1 ratio. The seedlings were maintained in a growth chamber at 23 ± 2 °C and at 70% relative humidity.

#### 4.1.2. Preparation of Aqueous Extracts from Coffee Leaves

The second pair of coffee leaves (from the apex to the base) was used to prepare the aqueous extracts. The leaves were oven dried at 50 °C for 48 h and then ground. The extraction was performed as described by Moreira et al. [39] with some modifications. A total of 100 mL of water was added to 10 g of leaves, and the extraction was performed by heating to 100 °C. Thereafter, the extracts were filtered on filter paper and freeze-dried.

#### 4.1.3. Determination of the Contents of Total Phenolics and Flavonoids

The Folin–Ciocalteu reagent was used to determine the total phenolic compound content. An aliquot of each extract (125 μL) was mixed with 625 μL of Folin–Ciocalteu reagent (diluted 1:10 in distilled water) and 500 μL of 4% (*w*/*v*) sodium carbonate in distilled water. After 2 h of incubation in the dark, the absorbance was measured in a spectrophotometer (750 nm). The total phenolic compound content was expressed as gallic acid equivalents (mg GA g^−1^), calculated using a curve constructed with concentrations ranging from 5 to 100 μg mL^−1^ [40]. The limits of detection and quantification used for this method were 3.45 mg g^−1^ and 10.45 mg g^−1^, respectively.

The determination of flavonoid content was performed using a 125 μL aliquot of extract, which was mixed with 375 μL of ethanol, 25 μL of 10% (*w*/*v*) aluminum chloride, 25 μL of 1 M potassium acetate and 700 μL of distilled water, totaling 1250 μL of reaction. After 30 min, the absorbance was measured in a spectrophotometer (425 nm). The analytical curve for total flavonoids was constructed using the quercetin solution as standard. The total flavonoid content was expressed as quercetin (Q) equivalents (mg Q g^−1^ extract) [41]. The limits of detection and quantification used for this method were 3.59 mg g^−1^ and 10.89 mg g^−1^, respectively.

#### 4.1.4. Determination of the Phenolic Compound Profile by High Performance Liquid Chromatography

The determination of the phenolic compound profile by high performance liquid chromatography was conducted using the following standard compounds for analysis: gallic acid, catechin, chlorogenic acid (5-caffeoylquinic acid), caffeic acid, vanillin, p-coumaric acid, ferulic acid, m-coumaric acid, coumarin, o-coumaric acid, rosmarinic acid, quercetin and trans-cinnamic acid. The samples were quantified using external standardization [42]. Analytical curves were constructed by triplicate injection of the standard solutions obtained by dilutions of the stock solution containing each standard at a concentration of 4 × 10^−5^ mol L^−1^. The analytical curves were obtained by linear regression considering the minimum correlation coefficient at 0.995. The identification of the analytes contained in the extracts was confirmed by the retention time and sample peaks in relation to the standards. The elution solvents used in the mobile phase were 2% acetic acid solution in water (phase A) and 70% methanol in 2% acetic acid solution in water (phase B). The detection wavelength was set at 271 nm. The identity of the analytes contained in the extracts was confirmed by the retention time and by the sample peaks in relation to the standards.

#### 4.1.5. Antioxidant Activity: Free Radical Scavenging Activity (DPPH) and Ferric Reducing Antioxidant Power (FRAP)

The free radical scavenging activity was evaluated by the 2,2-diphenyl-1-picrylhydrazyl (DPPH) radical scavenging method using the stable radical DPPH [43]. The extracts were prepared in microtubes with four different dilutions in triplicate to determine EC_50_. A 25 μL aliquot of each extract dilution was transferred to microtubes with 975 μL of a 0.06 mM solution of DPPH. The reduction in the DPPH radical was determined at 515 nm. The results were expressed in EC50, characterized as the value in which it expresses the amount of antioxidant needed to decrease its radical concentration by 50% [43,44]. The EC50 value is negatively related to the antioxidant activity, the lower the EC50 value, the higher the antioxidant activity of the tested sample.

The Fe-ion reducing power assay was performed with the leaf extracts using quercetin as the standard compound [10,45]. The FRAP reagent was prepared by mixing 300 mM sodium acetate buffer (pH 3.6); 10 mM 2,4,6-tri(2-pyridyl)-s-triazine (TPTZ) solution in 40 mM HCl; and 20 mM ferric chloride solution. Absorbance was carried at 593 nm, and ferrous sulfate heptahydrate was used as the standard.

The experimental design was completely randomized with two treatments (Mundo Novo and Iapar-59 cultivars), with six replicates and the experimental plot consisted of three coffee seedlings.

### 4.2. Production of Secondary Metabolites in Iapar-59 Coffee Leaves Inoculated with P. syringae pv. garcae

#### 4.2.1. Inoculation with *P. syringae* pv. *garcae*

Coffee seedlings of the cultivar Iapar-59 with four pairs of fully expanded leaves were used in this trial. A virulent isolate of *P. syringae* pv. *garcae* (obtained from the collection of microorganisms, Laboratory of Plant Bacteriology at the Federal University of Lavras) was used to inoculate the coffee seedlings. The bacterial suspension used as inoculum was prepared from cultures grown for 48 h on nutrient agar medium (0.5% peptone, 0.3% meat extract, 0.1% NaCl and 18 g agar in 1 L of distilled water), suspended in a saline solution (0.85% NaCl), and standardized in a spectrophotometer to contain approximately 5.1 × 10^9^ CFU.mL^−1^ (A600 = 0.8). The bacterial suspension was sprayed on the abaxial surface of the second pair of leaves. Control plants were inoculated only with saline solution. Plants were kept in a humid chamber for 24 h after inoculation. The collection of leaf samples was performed five days after inoculation. The samples were stored individually in aluminum foil, immediately immersed in liquid nitrogen (−196 °C) and then stored in an ultra-freezer at −80 °C until the preparation of the extracts and the analysis of the metabolites (total phenolic compounds, flavonoids, and reducing sugars).

#### 4.2.2. Quantification of Total Phenolic and Flavonoid Compounds, Reducing Sugars and Determination of Phenolic Profile in Leaves

Samples were obtained as described in Section 4.1.2. After extraction, the extracts were utilized to determine the total phenolic compound [40], flavonoid [41] and reducing sugar contents [46]. The phenolic profile was determined by high-performance liquid chromatography (HPLC) as reported in the Section 4.1.4.

The experimental design of experiment production of secondary metabolites in Iapar-59 coffee leaves inoculated with *P. syringae* pv. *garcae* was completely randomized with two treatments (inoculated and non-inoculated plants), six replicates and the experimental plot consisted of three coffee seedlings.

#### 4.2.3. Severity Disease in Mundo Novo and Iapar-59 Cultivars

Ten coffee seedlings from the cultivars Iapar-59 and Mundo Novo 376/4 were used in the pathogenicity test. The severity of BHB was determined using the diagrammatic scale proposed by Belan et al. [47], 20 days after inoculation with *P. syringae* pv. *garcae*.

### 4.3. Statistical Analysis

The original data of the total phenolic compounds, flavonoid and reducing sugar contents, free radical scavenging activity (DPPH), and ferric reducing antioxidant power (FRAP) were subjected to analysis of variance, and their means were compared by Tukey’s test at 5% probability.

## Figures and Tables

**Table 1:** Contents of total phenolic compounds (mg GA g^−1^ extract), flavonoids (mg Q g^−1^ extract), gallic acid (mg 100 g^−1^), chlorogenic acid (mg 100 g^−1^), and caffeic acid (mg 100 g^−1^) of aqueous extracts of coffee leaves from the cultivars Iapar-59 and Mundo Novo 376/4.

Cultivar	Phenolic Compounds	Flavonoids	Gallic Acid	Chlorogenic Acid	Caffeic Acid
Iapar-59	597.84 a	31.87 a	47.24 a	1.93 a	1487.42 a
Mundo Novo 376/4	394.54 b	17.11 b	27.14 b	1.51 a	975.11 b

Means followed by the same lowercase letter in each column do not differ by Tukey’s test (*p* < 0.05) (n = 6 biological replicates).

**Table 2:** Contents of reducing sugars (mg glucose g^−1^ extract), flavonoids (mg Q g^−1^ extract), total phenolic compounds (mg GA g^−1^ extract), gallic acid (mg 100 g^−1^), chlorogenic acid and caffeic acid (mg 100 g^−1^) in extracts of Iapar-59 coffee leaves inoculated and non-inoculated with *P. syringae* pv. *garcae*.

Treatments	Reducing Sugars	Flavonoids	Phenolic Compound	Gallic Acid	Chlorogenic Acid	Caffeic Acid	Vanillin
Inoculated	53.86 b	52.50 b	397.38 b	72.99 a	3.04 a	1697.29 a	20.19
Non-inoculated	89.29 a	120.19 a	755.52 a	47.24 b	1.93 b	1482.42 b	Nd

Means followed by the same lowercase letter in the column do not differ statistically by the Tukey test (*p* < 0.05). Nd: compound not detected.

## Data Availability

The data are available from the corresponding author upon reasonable request.
